# The Potential Role of Gut Microbiota in the Prevention and Treatment of Lipid Metabolism Disorders

**DOI:** 10.1155/2020/8601796

**Published:** 2020-09-14

**Authors:** Yan-Jun He, Chong-Ge You

**Affiliations:** Laboratory Medicine Center, Lanzhou University Second Hospital, No. 82 Cuiyingmen Lanzhou, Lanzhou 730030, Gansu, China

## Abstract

Due to changes in lifestyle, diet structure, and aging worldwide, the incidence of metabolic syndromes such as hyperlipidemia, hypertension, diabetes, and obesity is increasing. Metabolic syndrome is considered to be closely related to cardiovascular disease and severely affects human health. In recent years, researchers have revealed that the gut microbiota, through its own or interacting metabolites, has a positive role in regulating metabolic syndrome. Therefore, the gut microbiota has been a new “organ” for the treatment of metabolic syndrome. The role has not been clarified, and more research is necessary to prove the specific role of specific strains. Probiotics are also believed to regulate metabolic syndromes by regulating the gut microbiota and are expected to become a new preparation for treating metabolic syndromes. This review focuses on the regulation of lipid metabolism disorders by the gut microbiota through the effects of bile acids (BA), short-chain fatty acids (SCFAs), bile salt hydrolase (BSH), and genes such as ABCG5 and ABCG8, FXR, NPC1L, and LDL-R.

## 1. Introduction

With the aging of the population, the incidence of metabolic syndromes such as hyperlipidemia, hypertension, and diabetes has progressively increased. Metabolic diseases are associated with an increased risk of mortality in the elderly, and in particular, hyperlipidemia is an important risk factor for cardiovascular disease [1], leading to cardiovascular diseases such as atherosclerosis and coronary heart disease, which seriously endanger health. Yusuf et al. [2] conducted large standardized case-control studies in 52 countries around the world and found that the incidence of dyslipidemia, smoking, hypertension, diabetes, abdominal obesity, and psychosocial factors is associated with an increased risk of heart disease in all regions of the world, but edible fruits and vegetables, regular physical exercise, and avoiding smoking can reduce the relative risk of myocardial infarction, among which smoking and dyslipidemia are the two most important risk factors. Previous studies have found that some *Lactic Acid Bacteria* can reduce cholesterol in high-fat diet animals [3], improve insulin resistance and obesity [4], and alleviate the damage to the intestinal mucosal barrier [5]. In the case of abnormal lipid metabolism, improving the structure of the gut microbiota may affect the physiological state of the host.

Gut microbiota, the normal flora of the human intestinal tract, are numerous and abundant. *Bifidobacterium* genus and *Lactobacillus* genus can synthesize vitamins and essential amino acids that are necessary for human growth and development, and participate in the metabolism of sugars and proteins, which have an important role in human health. Gut microbes are a vast and complex system of the human body that contain 100 times more genes than in the human body [6]. The gut microbiota is mainly composed of four phyla, namely, *Firmicutes*, *Bacteroidetes*, *Actinobacteria*, and *Proteobacteria* [7]. Among them, *Bacteroides*, *Bifidobacterium*, *Eubacteria*, *Clostridium*, *Peptococcus*, *Peptostreptococcus*, and *Rumenococcus* are predominant, and *Escherichia*, *Enterobacter*, *Enterococcus*, *Klebsiella*, *Lactobacillus*, and *Proteus* are subdominant genus [8].

The way microorganisms affect human metabolism depends not only on their quantity but also the metabolites that interact with the host or themselves [9]. For example, *Lactobacillus paracei* and *Escherichia coli* inhibit lipid secretion by enterocytes increasing intracellular fat storage and enhancing lipid catabolism, respectively [10]. *Lactobacillus rhamnosus* can enhance the presence of another *Lactic Acid Bacterium Streptococcus thermophilus*, and the symbiotic relationship between the two and their production of metabolites (such as SCFA) enables the host intestinal cells to more efficiently use lipids in the diet, thereby reducing lipid content [11]. A recent study showed that feeding high-fat mice with *Lactobacillus plantarum* LC27 and *Bifidobacterium longum* LC67 can regulate the activation of NF-*κ*B and AMPK by inhibiting the production of lipopolysaccharides from the intestinal microbial flora, reducing liver steatosis, obesity, and colitis [12]. Early studies have shown that changes in the structure of the gut microbiota are related to some abnormal lipid metabolism, such as atherosclerosis, obesity, and diabetes [5–8]. In this review, we mainly introduce the role of gut microbes in regulating dyslipidemia and the underlying mechanism of this regulation and propose a microbial pathway for the treatment of lipid metabolism disorders.

## 2. Relationship between Gut Microbiota, Probiotics, Prebiotics, and Hyperlipidemia

### 2.1. Gut Microbiota and Hyperlipidemia

Gut microbiota is closely associated with disorders of lipid metabolism and even affect the occurrence of cardiovascular disease. In a six-year follow-up experiment by Framingham Study, a longitudinal prospective study of coronary heart disease, elevated serum cholesterol levels were considered to be bound up with the development of coronary heart disease [13]. The intestinal microbiome is a biomarker for cardiac metabolic disorders. *Prevotella*, *Bacteroides*, *Clostridium*, and *Faecalibacterium* show characteristic changes in patients with lipid metabolism disorders [9]. Kazuyuki et al. [14] found that plasma and liver cholesterol levels of Apo-E-deficient mice lacking intestinal microbiota were significantly increased, but the formation of atherosclerotic lesions was markedly reduced, which may be related to the weakening of the lipopolysaccharide-mediated inflammatory response, and the gut microbiota may promote atherosclerosis. Martinez-Guryn et al. [15] found that the small intestinal microflora affect the absorption of lipids in the intestine and regulates lipid metabolism by regulating the digestion and absorption process of intestinal epithelial cells and this process is related to the action of specific bacterial strains. This evidence further correlates the gut microbiota with lipid metabolism.

Diet is a key factor in the formation of the gut microbiota [16] and the intestinal microbiota can quickly respond to changing diets [17], affecting lipid absorption. High-fat diets lead to elevated serum and liver TG levels, which may be related to increased levels of de novo fat production and TG synthesis and decreased expression of genes related to fatty acid oxidation [18], and high-cholesterol diets can reduce the diversity of gut microbiota [19]. In addition, aging can cause changes in the composition of the gut microbiota [15]. Zeng et al [20] were the first to demonstrate that short-term (2 months) dietary restrictions in elderly mice can restore the elderly dysfunctional gut microbiota to young levels. This finding helps to improve the incidence of age-related diseases. Gender differences in fat metabolism have also been reported to be related to the composition of the intestinal microbiota. After oral probiotics, female serum TG and LDL-C decreased more markedly than in men [21, 22]. Haro et al. [23] found a correlation between gut microbiota and gender and body mass index in a prospective, randomized, open, and controlled trial. Jingyuan Fu et al. [24] studied 893 subjects from the Life Lines-DEEP population cohort and found that 34 bacterial groups are related to body mass index and blood lipids. Intestinal microbes may play an important role in the changes in body mass index and blood lipid levels, which is independent of age, sex, and host genetics. A meta-analysis found that, in terms of BMI, the reduction in TC and LDL-C was almost the same between normal and obese people. However, compared with short-term (4 weeks) intervention, long-term (>4 weeks) probiotic intervention can effectively reduce serum LDL-C and triglycerides [25].

It can be seen that the role of the gut microbiota is closely related to age, gender, body mass index, intervention time, and dosage. These contradictory results propose that we should individualize the treatment of lipid metabolism through the gut microbiota. In short, these observations provide novel avenues and insights for validation and follow-up research, while revealing the importance of holding a healthy diet to prevent the development of lifestyle-related diseases.

### 2.2. Probiotics, Prebiotics, and Hyperlipidemia

For the intervention of lipid metabolism in the body, many people have gradually realized the harm of long-term drug treatment, so they have paid more attention to the intervention of lifestyle. The diet is regarded as the basis to treat dyslipidemia, and exercise is also an important first-line intervention for dyslipidemia. Recent studies have shown that edible probiotics have a potential effect on lipid metabolism.

Probiotics are living microorganisms that, when given in sufficient amounts, have a beneficial effect on the host by altering the balance of known flora in the intestine [26]. Probiotics are increasingly important for health benefits, but one obstacle to using new probiotic cultures is their safety [3]. Therefore, it is necessary to strictly check the biosafety and health benefits of probiotics before using them. Human probiotic microorganisms belong mostly to the following genera: *Lactobacillus*, *Bifidobacterium*, *Lactococus*, *Streptococcus*, and *Enterococcus* [27]. At present, the most used probiotics mainly include *Bifidobacteria* and/or lactobacilli, as well as other *Lactic Acid Bacteria*, such as lactococci and streptococci [28].

Probiotics can increase the peristalsis of the intestine [29], reduce the residence time of feces in the intestine, and prevent harmful substances from getting into the body from the intestine. Most studies have shown that the intake of probiotics can reduce serum TC and TG [11, 12, 21, 22, 29–34], and only a small part can decrease LDL [12, 21, 30, 32, 34–37] and increase HDL [12, 16, 32, 34, 35, 37]. The intake of probiotics will also change the structure of the gut microbiota. Most of them can increase the proportion of beneficial bacteria such as *Lactobacillus* and *Bifidobacterium* [11, 29–31, 33, 35, 38, 39] (Table 1). The gut microbiota is mainly controlled by *Firmicutes* and *Bacteroides* [9]; therefore increases in one almost always imply reductions in the other. In patients with mild hyperglycemia and dyslipidemia, one week after oral administration of *Bifidobacterium bifidum* TMC 3115 strain, the level of the *Firmicutes* phylum increased and that of the *Bacteroides* phylum decreased [21].

Probiotics may reduce cholesterol by promoting fatty acid oxidation and reducing fatty acid synthesis in the liver, promoting intestinal development and digestion, and balancing gut microbiota in the small intestine, reducing fat accumulation [31]. However, some experiments have shown that probiotics may not always have a positive effect on gut microbiota. Ataie-Jafari et al. [41], by giving yogurt containing two probiotics (*Lactobacillus acidophilus* and *Lactobacillus*) to patients with hypercholesterolemia, found that compared with ordinary yogurt, except for significantly lowering total serum cholesterol, there were no other changes. Mazloom et al. [42] found that probiotics had no significant effect on blood lipid- and blood glucose-related parameters in patients with type 2 diabetes. Different probiotics have different effects on metabolism, and this result may depend on the strain, dosage, and ingredients used to produce a particular probiotic product [27].

Prebiotics are nondigestible food components used by intestinal microbes. They can selectively stimulate the growth and/or activity of one or more beneficial bacteria and have a beneficial effect on the host [43]. For example, after feeding high-fat diet mice supplemented with 1% grape polyphenols for 12 weeks, the intestinal microbial community structure significantly changed, the amount of *Akkermansia muciniphila* in the intestine increased, the ratio of *Firmicutes* to *Bacteroidetes* decreased [44], and the proliferation of *Akkermansia muciniphila* depends on its baseline abundance [45]. In addition, the increased colonization of *Akkermansia muciniphila* in the intestinal tract protects mice from the excessive production of chylomicrons and VLDL induced by acute lipid load, helps to maintain the homeostasis of lipid metabolism in the host, and alleviates the symptoms of metabolic syndrome [46]. Alissa C. and others were the first to give oligofructose-enriched inulin to overweight and obese children. It was found that serum triglycerides in the prebiotic group were significantly reduced, and the number of *Bifidobacteria* and *Actinomyces* increased [47], thereby improving obesity outcomes of overweight/obese children. Prebiotic treatment increases plasma intestinal peptide concentration (glucagon-like peptide 1 and YY peptide) [48] and the expression of intestinal antimicrobial peptide regenerating islet-derived 3-gamma, which is a key protein in the transformation of intestinal epithelial cells. It also regulates the structure of the intestinal flora, improves the balance of the intestinal environment, and helps strengthen the intestinal barrier induced by prebiotics [49] and regulate human obesity and metabolic syndrome. Previous studies have also found that prebiotics can improve blood glucose regulation disorders (glucose tolerance and insulin resistance) in db/db mice [50].

These findings provide the basis for probiotics and prebiotics as a well-tolerated, easy-to-use natural cholesterol-lowering supplement, confirming that probiotics and prebiotics activate metabolic pathways and enhance overall health. Therefore, supplementing probiotics and prebiotics can be useful in preventing hyperlipidemia function and may reduce risk factors for cardiovascular disease.

## 3. Proposed Mechanisms of Gut Microbiota on Hyperlipidemia

### 3.1. Biological Mechanisms of Gut Microbiota to Reduce Cholesterol

The gut microbiota is associated with the metabolism of two types of cholesterol: one is de novo cholesterol synthesized from the diet or liver, and the other is cholesterol from tissues and bile acids synthesized by cholesterol [51]. There are several possible mechanisms by which gut microbiota can lower cholesterol.

Cholesterol is bound to the cell surface, and the bound cholesterol is not easily absorbed by the enterohepatic circulation, resulting in increased cholesterol excretion [35]. Another study showed that *Lactobacillus acidophilus* can also reduce cholesterol by assimilating cholesterol to the cell membrane, and its assimilation is impacted by the amount of bile [52].

In addition to clearing cholesterol directly from the systemic circulation, cholesterol can also be converted into bile acids in advance [53]. Bile acid is an important component of bile and plays a significant role in fat metabolism. About 95% of bile acids are reabsorbed in the distal ileum and returned to the liver through the enterohepatic circulation, and the remaining 5% of bile acids are excreted in the feces. Cholesterol 7 alpha-hydroxylase(CYP7A1) is used to convert cholesterol in the liver into bile acids and excreted in feces, which are the main pathways of cholesterol catabolism [54], whereas sterol-27 hydroxylase (encoded by CYP27A1) and CYP7B1 is mainly involved in the alternative pathway [55]. Under the action of the gut microbiota, the primary BA is converted to secondary BA. The production of secondary bile acids can be mediated by bile salt hydrolase [56], high-fat diet, and high expression of bile salt hydrolase (BSH) genes that cause fecal deoxy cholic acid (DCA) increases [17]. Chen et al. [29] found that the bile acid concentration in the stool of the *Lactobacillus rhamnosus* hsryfm 1301 group was considerably higher than that in the control group (*p* < 0.05), confirming this view. *Lactobacillus rhamnosus* hsryfm 1301 was screened from gut samples taken from individuals living in the Bama Longevity Village, Guangxi Province in China, and has the ability to lower blood lipids, which reduces the concentration of cholesterol by inducing the deconjugation and increased precipitation of bile acids in the intestinal lumen, thereby increasing its fecal excretion.

**Figure 1:**
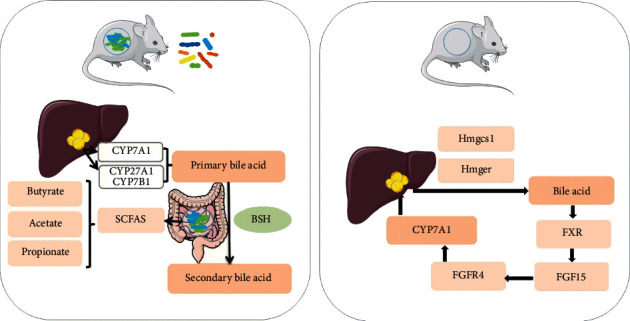
The metabolic pathways of gut microbiota in conventional and germ-free mice.

In turn, elevated DCA levels may cause microbial disturbances on the animal-based diet, inhibiting the growth of *Bacteroidetes* and *Firmicutes* [17]. Bile acid synthesis is regulated by cholesterol enzymes. HMG-CoA synthase 1 (Hmgcs1) and HMG-CoA reductase (Hmgcr), which are involved in cholesterol synthesis, are downregulated in the liver of sterile mice, so there is the lack of intestinal microbiota bile acid synthesis in hypercholesterolemia mice [14]. Öner et al. [57] studied the cholesterol-lowering mechanism of *Lactic Acid Bacteria* and *Bifidobacteria* and showed that only probiotics containing the bile salt hydrolase gene can reduce cholesterol, and increased bile salt hydrolase activity leads to increased bile excretion into feces [35]. BSH is widely distributed in *Bacteriodetes*, *Clostridium*, *Lactobacillus*, *Enterococcus*, *Streptococcus*, *Listeria*, and *Bifidobacterium* [58]. BSH-active probiotics can increase the deconjugation of bile salts in enterohepatic circulation, leading to increased levels of deconjugated bile acids in the circulation, and the solubility of bile acids decreases after deconjugated and is absorbed in the intestinal tract and, then, excreted in feces [22]. Cholesterol is, then, used to synthesize de novo bile acids in the body, thereby reducing serum cholesterol [59]. However, BSH activity leads to an increase in the production of secondary bile acids, which may have a negative impact on human health. The increase in their concentration in the intestine may cause intestinal inflammation [60] and may even promote intestinal cancer [61] (Figure 1).

In conventional mice, cholesterol is converted into bile acids through the main pathway that CYP7A1 participates in and another pathway that CYP27A1 and CYP7B1 participate in. Under the action of the gut microbiota, primary bile acids are transformed into secondary bile acids. This process can also be mediated by BSH. Some probiotics reduce the cholesterol concentration by inducing the deconjugation of bile acids in the intestinal lumen and increasing precipitation. In addition, the gut microbiota can produce SCFA (especially, butyrate, acetate, and propionate), blocking the synthesis of liver cholesterol. In germ-free mice, the expression of FXR is upregulated. Bile acid acts as a ligand to bind and activate FXR, resulting in an increase in FGF15. FGF15 binds to FGFR4 and inhibits the expression of CYP7A1 gene, which affects cholesterol metabolism. In addition, Hmgcs1 and Hmgcr, which are involved in cholesterol metabolism, are downregulated in the liver of sterile mice, leading to increased liver cholesterol levels. Lack of gut microbiota will affect the metabolism of cholesterol, reduce the production of bile acids, and lead to elevated cholesterol.

In addition, the gut microbiota can increase the content of short-chain fatty acids (SCFAs) in the intestine, blocking the synthesis of liver cholesterol, transfer plasma cholesterol to the liver, and reduce lipid levels in the blood [11, 33]. SCFAs are produced from indigestible carbohydrates (including dietary fiber, resistant starch, and oligosaccharides) in the intestine and are absorbed from the colon to the liver and surrounding tissues [24]. Previous studies have shown that the addition of *Lactobacillus* probiotics to the diet can induce the production of SCFA by regulating the intestinal microbiota [62–64], and many commensal bacteria in the gut produce SCFAs, especially butyrate, acetate, and propionate, which have proved to reduce the risk of gastrointestinal diseases [65]. Acetic acid and propionate are mainly produced by *Bacteroides*, and propionate can especially reduce the synthesis of cholesterol in the liver and improve lipid metabolism [5]. A high-fat diet can substantially reduce the content of butyric acid, affect intestinal metabolism, and increase the incidence of inflammation [66]. Elevated levels of *Lactic Acid Bacteria* in the intestine can promote the content of SCFAs, lactic acid, and essential amino acids (tyrosine, phenylalanine, leucine, isoleucine, valine, and lysine), which proves the effect of specific *Lactobacillus* on intestinal flora [67]. Sodium propionate treatment can reduce the concentrations of butyric acid and valeric acid to normal levels in HFD-fed mice, increase the number of *Bacteroides*, reduce the number of *Lactic Acid Bacteria* and *Firmicutes*, restore serum LPS concentrations to normal levels, regulate the intestinal bacteria group, and can effectively resist lipid disorders caused by HFD [68].

Most of the current treatment strategies for hyperlipidemia are a combination of lipid-lowering drugs and lifestyle changes, especially diets that limit fats and carbohydrates to reduce plasma lipid levels. The role of the gut microbiota provides early evidence for replacement or supplementation of existing treatments.

### 3.2. Gut Microbiota Affects Lipid Metabolism-Related Genes and the Signaling Pathway

Although lipid metabolism disorders are linked to lifestyle and other related metabolic diseases, such as overweight, obesity, or diabetes, some hyperlipidemia are caused by genetic changes [16]. Junjie Qi and his colleagues used Illumina GA short-read-based sequencing of total fecal DNA from 124 individuals from Europe to create 3.3 million nonredundant microbial genes, which contained the vast majority of human intestinal microorganism gene [7].

One of the main goals of reducing the risks of cardiovascular disease is LDL-C [22]. Kasahara et al. [14] found that compared with conventional mice, the expression of the LDL receptor (LDL-R) in the liver of germ-free (GF) mice is suppressed, the LDL-R gene is regulated by SREBP-2 (sterol regulatory element-binding transcription factor 2), and the expression of SREBP-2 is downregulated in germ-free mice and causes elevated plasma LDL levels, suggesting a beneficial effect of the gut microbiota. Shen et al. [46] found that *Akkermansia muciniphila* protects the host from acute and chronic hyperlipidemia by enhancing the expression of low-density lipoprotein receptors and Apolipoprotein E(APO-E). It can be seen that LDL-R signaling is essential in mediating the effect of reducing triglycerides in mice, decreasing the risk of cardiovascular disease. Stepankova et al. [69] found that germ-free (GF) conditions accelerated the development of atherosclerosis in Apo-E-deficient mice fed a low-cholesterol diet. However, the difference in atherosclerotic plaques in GF and traditional Apo-E-deficient mice on a high-cholesterol diet is not obvious. Changing the composition of bacteria through a specific diet or regulating the formation of microbial metabolites may have a long-term impact on the development of cardiovascular diseases.

**Figure 2:**
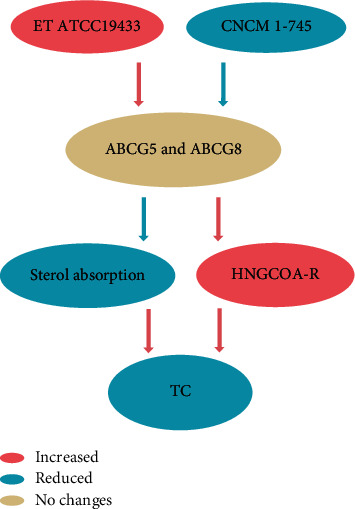
Different probiotics have the same effect on cholesterol through ABCG5 and ABCG8.

The ABCG5/8 gene encodes a heterodimer transporter that promotes the efficient secretion of cholesterol from liver cells to bile, reducing cholesterol levels. *Enterococcus faecalis* ATCC19433 increases the content of ABCG5 and ABCG8, which can reduce the cholesterol content of the liver and the weight of the liver. The mechanism may be that ABCG5 and ABCG8 can inhibit the absorption of sterols and promote the excretion of biliary sterols [38]. Yet, another study is the opposite. F. Briand fed hypercholesterolemia hamsters with *Saccharomyces boulardii* CNCM I-745, and CNCM I-745 significantly reduced the expression of ABCG5 and ABCG8 in hypercholesterolemia and increased the expression of HMG CoA-R gene (encoding a rate-limiting enzyme of cholesterol synthesis). However, total cholesterol in plasma decreased significantly and total stool cholesterol increased (*P* < 0.05) [40]. In general, although the pathways are different, the overall effect is the same, and the specific mechanism of specific strains needs further research (Figure 2).

*Enterococcus faecalis* ATCC19433 increases the content of ABCG5 and ABCG8, and ABCG5 and ABCG8 can inhibit the absorption of sterols and promote the excretion of biliary sterols. *Saccharomyces* boulardii CNCM I-745 significantly reduced the expression of ABCG5 and ABCG8 in hypercholesterolemia and increased the expression of HMG CoA-R gene. Both total cholesterol in plasma decreased significantly.

Another important regulator of triglyceride and cholesterol homeostasis is the FXR [70]. The Farnesoid-X-Receptor (FXR) is a nuclear receptor [71], and bile acid acts as a ligand to bind and activate the FXR, leading to the release of fibroblast growth factor 15 (FGF15) [58]. Kasahara et al. found that the lack of intestinal microbiota in Apo-E-deficient mice caused significant increases in plasma and liver cholesterol levels, which may be related to the upregulation of FXR expression in the terminal ileum of GF mice. The upregulation of the FXR leads to an increase in the expression of FGF15, and FGF15 leads to the upregulation of the expression of fibroblast growth factor receptor 4 (FGFR4) in the liver. FGF15 binds to FGFR4 in hepatocytes and inhibits the expression of CYP7A1 gene, reduces the total bile acid content in the liver, and leads to an increase in liver cholesterol levels [14]. Therefore, the lack of intestinal microbiota changes the distribution of bile acids, which may stimulate the liver and intestine FGF15/FGFR4 axis, reduce the expression of liver CYP7A1, the synthesis of bile acids, and the catabolism of cholesterol, leading to an increase in liver cholesterol levels. Previous studies have found that probiotics downregulate FXR, leading to increased liver bile acid synthesis in mice [72]. An intestine-selective FXR inhibitor can improve obesity and metabolic syndrome in HFD-induced obese mice [73]. From the abovementioned content, it can be inferred that inhibiting the intestinal FXR may be a reasonable therapeutic strategy for the treatment of human metabolic disorders (Figure 1).

The gut microbiota alters fat storage by regulating the lipoprotein lipase (LPL) inhibitor Fiaf [74]. Fiaf is related to the deposition of triglycerides in adipocytes, and the peroxisome proliferator-activated receptor *γ*(PPAR) is involved in microbially induced Fiaf expression. The increase in Fiaf is related to the increase in gene expression that regulates cholesterol metabolism [65]. Decreased expression of PPAR-regulated genes in the intestine after colonization by *Lactobacillus paracasei* and *E*. *Coli* is involved in intracellular levels of free fatty acids (FFAs) [10]. FFAs are lipid substances released from adipose tissue and various cells after lipid breakdown. Javier Rodríguez-Carrio et al. [75] demonstrated that the intestinal abundance of *Akkermansia muciniphila* is the principal predictor of serum total FFA levels and is inversely connected with total FFA and the proinflammatory factor IL-6.

Niemann-Pick C1-Like 1 (NPC1L1) is a transmembrane protein that participated in cholesterol absorption [53]. Hepatocyte nuclear factor 4 (HNF4a) is a cholesterol-dependent signal that plays a vital role in the regulation of NPC1L1. Silvia Falcinelli and others fed *L*. *rhamnosus lactobacillus* to hyperlipidemia model mice which reduced the expression of HNF4a gene and, then, downregulated NPC1L1, resulting in a decrease in cholesterol content. *L*. *rhamnosus* also affects triglycerides hydrolysis through the regulation of MGLL and the gene encoding transmembrane protein (FIT2). This indicates that probiotics reduce the transcription of genes related to cholesterol metabolism, but the level of transcription gets nothing to do with dietary fat content [76]. A previous study showed that, after the addition of probiotics, the MGLL gene expression level in the gallbladder increased, and the triglycerides content decreased, further proving the role of probiotics [11].

## 4. Conclusions

More and more studies have discovered the importance of gut microbiota and probiotic intake in lipid metabolism disorders. Changes in the structure of gut microbiota can affect atherosclerosis, obesity, and diabetes. Researchers have discovered many mechanisms about gut microbiota, including biological mechanisms, genes, and signaling pathways. In addition, probiotics have a potential role in the gut microbiota and can regulate lipid metabolism, obesity, and insulin resistance in the body to varying degrees. In future research, more attention should be given to the research of specific strain mechanisms to make the treatment more individual. Therefore, the use of probiotics in humans needs to be validated in the long-term, large-scale population.

## Figures and Tables

**Table 1:** Recent studies on the effects of probiotics on hyperlipidemia.

Author, year	Model (human/animal)	Gender	Probiotics	Dosage and duration	Changes in gut microbiota	Lipid changes
Falcinelli et al., 2015 [11]	Zebrafish	Female and male	*Lactobacillus rhamnosus*	1 × 10^6^ CFU/ml 18 hours	Increased *Firmicutes*, *Lactobacillus*, and *Streptococcus thermophilus*; reduced *Actinomyces*	Reduced TC and TG
In Kim et al., 2019 [12]	C57BL/6 mice	Male	*Lactobacillus plantarum*LC27, *Bifidobacterium longum*LC67	1 × 10^9^ CFU/ml 4 weeks	Reduced *Firmicutes* and *Proteobacteria*	Reduced TC and LDL-C; increased HDL-C
Wang et al., 2018 [21]	Human	No description	*Bifidobacterium bifidum* TMC3115	3 × 10^10^ CFU/mL 3 weeks	Increased *Firmicutes*; reduced *Bacteroides*	Reduced TC and LDL-C
Costabile et al., 2017 [22]	Human	34 females, 15 males	*Lactobacillus plantarum* ECGC 13110402	2 × 10^9^ CFU/ml 12 weeks	No significant differences	Reduced TC and TG; increased HDL-C
Shin et al., 2018 [30]	Sprague Dawley rats	Male	A probiotic formulation containing 2 *Lactobacillus* strains, 3 *Bifidobacterium*, and *Streptococcus thermophilus* ST3	1 × 10^7^ CFU/ml 8 weeks	Increased *Bacteroides*, *Lactobacillus*, and *Bifidobacterium*; reduced *Firmicutes*	Reduced TC, TG, and LDL-C
Salazar et al., 2019 [31]	C57BL/6 mice	Male	*B. animalis* IPLA R1 strain	5 × 10^8^ CFU/ml 10 days	Increased *Bifidobacterium*	Reduced TG
wang et al., 2017 [32]	Chicks	Male	*Lactobacillus johnsonii*	1 × 10^6^ CFU/ml 42 days	Increased *Bacteroides* and *Lactobacillus*; reduced *Escherichia coli*	Reduced TG and LDL-C increased HDL-C
Chen et al., 2015 [29]	Wistar rats	Male	*Lactobacillus rhamnosus* hsryfm 1301	10^9^ CFU/ml 28 days	Increased *Bifidobacterium*, *Lactobacillus*, *Bacteroides*, and *Enterococcus*; reduced *Escherichia coli* and *Clostridium*	Reduced TC and TG
Park et al., 2018 [34]	C57BL/6JTacN mice	Male	*Lactobacillus rhamnosus* strain BFE5264	1 × 10^10^ CFU/ml 9 weeks	Increased *Mucispirillum*	Reduced TC and LDL-C; increased HDL-C
lyea et al, 2017 [35]	BALB/*c* mice	Male	*Lactobacillus fermentum* FTDC 8312	1 × 10^9^ CFU/ml 7 weeks	Increased *Akkermansia*, *Desulfovibrio*, *Oscillospira*, and *Lactobacillus*	Reduced TC and LDL-C; increased HDL-C
Zhu et al., 2019 [38]	C57BL/6J mice	Male	*Enterococcus faecalis* ATCC19433	5 × 10^9^ CFU/ml 4 weeks	Increased *Lactobacillus*, *Bifidobacterium*, *Akkermansia*, and *Bacteroides*; reduced *Firmicutes*	Reduced TC and LDL-C
Briand et al., 2019 [40]	Golden Syrian hamsters	Male	*Saccharomyces boulardii* CNCM I-745	3g/kg 39 days	Increased *Proteobacteria* and *Lentispharerae*; reduced *Firmicutes*, and *Tenericutes*	Reduced TC; reduced HDL-C at 21 days
Liu et al., 2017 [36]	Immature kunming rats	Male	*Lactic Acid Bacteria* 96	1 × 10^9^ cfu/mL 5 weeks	Reduced *Escherichia coli*	Reduced TG, TC, and LDL-C
Ichim et al., 2016 [37]	C57BL/6J mice	No description	DBR:a proprietary blend of 9 probiotic organisms and 10 digestive enzymes	65 *μ*g/ml 8 weeks	Increased *Lactobacilli*	Reduced LDL-C; increased HDL-C

CFU: colony-forming units; TC: total cholesterol; TG: triglyceride; HDL-C: high-density lipoprotein cholesterol; LDL-C: low-density lipoprotein cholesterol.
